# The microenvironment of classical Hodgkin lymphoma: heterogeneity by Epstein–Barr virus presence and location within the tumor

**DOI:** 10.1038/bcj.2016.26

**Published:** 2016-05-06

**Authors:** R Wu, A Sattarzadeh, B Rutgers, A Diepstra, A van den Berg, L Visser

**Affiliations:** 1 Department of Pathology and Medical Biology, University of Groningen and University Medical Center Groningen, Groningen, The Netherlands; 2 Department of Biochemistry/Open laboratory for Tumor Molecular Biology, Shantou University Medical College, Shantou, China; 3 Department of Hematology and Oncology, 2nd Affiliated Hospital of Shantou University Medical College, Shantou, China

Classical Hodgkin lymphoma (cHL) is characterized by a low number of malignant cells, called Hodgkin Reed–Sternberg (HRS) cells, surrounded by an abundant infiltrate of immune cells. Presence of T helper (Th)2 and regulatory T cells (Treg) in the infiltrate was shown by staining of T-cell subtype specific transcription factors in cHL tissue samples^1^ and by expression of Th2- and Treg-specific cytokines in sorted T-cell subsets.^2^ However, one study revealed a predominant Th1 subpopulation in the microenvironment of cHL based on expression of CXCR3 by flow cytometry.^3^ Differences between these studies may potentially be explained by heterogeneity between Epstein–Barr virus (EBV)-positive and -negative cHL cases and by differences in cell composition in relation to proximity of tumor cells.

In this study, we compared the composition of the reactive infiltrate of EBV+ (*n*=7) and EBV− cHL (*n*=7) with each other and with reactive lymph nodes (RLNs) for 46 specific cell types (Supplementary Table S1). In addition, we made a distinction between cells that are located in the close vicinity of tumor cells and cells that are further away. The characteristics of the study cohort has been described in Table 1. The gender distribution and age range of the HL patients are comparable with the RLN cohort. There is no significant difference in ages between the EBV+ and EBV− patients. The majority of the HL patients have nodular sclerosis (NS) histology, within the EBV+ cHL group relatively more mixed cellularity (MC) cases are found.

We did not find any significant differences between HL and RLN in the main cell populations (B cells, T cells, natural killer (NK) cells and macrophages)(Supplementary Table S2). In contrast to previous studies using immunohistochemistry (range 3–57%),^4^ we observed relatively low percentages of macrophages. This might be caused by a less optimal isolation of macrophages from fresh tissues, resulting in an underrepresentation of these cells in our flow analysis.

Analysis of different T-cell subpopulations revealed significantly more GITR+CD25+ and FoxP3+CD25+ Treg cells in the CD4+ T-cell subset in both EBV+ and EBV− cHL compared with RLNs (Figures 1a and b). This increase in Treg cells is consistent with a previously published study.^5^ We observed no significant difference for EBV+ and EBV− cHL in the percentage of Tregs, suggesting that suppression of the immune response is important in both EBV+ and EBV− cHL. FoxP3+CD25+CD4+ Tregs were upregulated in EBV+ HL cases by immunohistochemistry.^6^ Moreover, upregulation of messenger RNA (mRNA) levels for Treg-associated markers and cytokines has also been described in EBV+ cHL compared with RLN and EBV− cHL, especially for T regulatory 1 cells, that produce interleukin-10.^7^ Together these findings indicate that Tregs are important in all cHL cases, albeit more pronounced in the microenvironment of EBV+ HL.

The percentage of CD56+CD16+ NK cells was significantly lower in EBV− cHL compared with RLN and EBV+ cHL (Figure 1c). In addition, we observed significantly more CD69+, granzyme B+ and TIA-1+ cells in the CD8+ T-cell subset in EBV+ cHL compared with RLN and EBV− cHL (Figures 1d–f). Thus, EBV status has a positive correlation with the percentage of NK cells and activated CD8+ T cells. This suggests that the presentation of EBV-derived antigenic peptides within human leucocyte antigen class I molecules triggers antiviral immune responses. The number of NK cells can increase upon recognition of virus by toll-like receptors. The higher numbers of cytotoxic CD8+ T cells in EBV+ cHL indicates a Th1 response as Th1 cells stimulate a cellular-immune response and activate CD8+ T cells. In contrast to the findings of Greaves *et al.*,^3^ we did not find significant increases in the percentage of CXCR3+ Th1 cells within the CD4+ cells in either EBV+ or in EBV− cHL.

The combined presence of Tregs and activated CD8+ T cells raises the questions on how they can coexist in cHL and where they are located in respect to the tumor cells. To answer these questions, we used CD26 to discriminate between T cells in the area of the HRS cells, that is, the CD26− T cells, and the T cells that are not in close contact with the HRS cells, that is, the CD26+ T cells.^2^ To confirm the relevance of using CD26 as a marker, we first stained CD26 in the cHL tissue sections. In the NS and lymphocyte-rich subtypes of cHL, CD26− cells were indeed located in the close vicinity of tumor cells, whereas CD26+ cells were not observed in the tumor cell areas. In MC, the CD26+ cells were much more dispersed and were also found close to the tumor cells. Based on these staining results, we excluded the MC cases from the subsequent analyses.

We found significantly more CD69+ Th cells in the CD26− T-cell subset indicating that these cells are more abundant in the tumor cell area (Figure 1g). This suggests triggering of an immune reaction in these cells because CD69 is an early activation marker. A similar pattern was observed in EBV+ as well as EBV− cHL. Furthermore, we also observed more FoxP3+-suppressive Th cells in the tumor cell area in both EBV+ and EBV− cHL (Figure 1h). These findings are consistent with increased mRNA levels of Treg-associated genes (*CTLA4*, *IL2RA*, *TNFRSF4* and *CCR4*) in CD4+CD26− T cells in cHL compared with RLN.^2^ So, the Tregs reside in the direct vicinity of the HRS cells and might inhibit an effective antitumor immune response. There were less CD25+ CD8+ cells around the tumor cells than outside the tumor cell area (Figure 1i). So, although activated CD8+ cells are present in the microenvironment of cHL, these cells are not present in the tumor-cell-rich areas and thus not effective.

In conclusion, we showed that the main difference in the composition of the microenvironment between EBV+ and EBV− cHL patients is caused by increased numbers of both CD8+ T cells and NK cells. High numbers of CD4+ Tregs directly surrounding HRS cells imply that they have an important role in the failure to induce an effective immune response against the HRS cells of cHL.

## Figures and Tables

**Figure 1:**
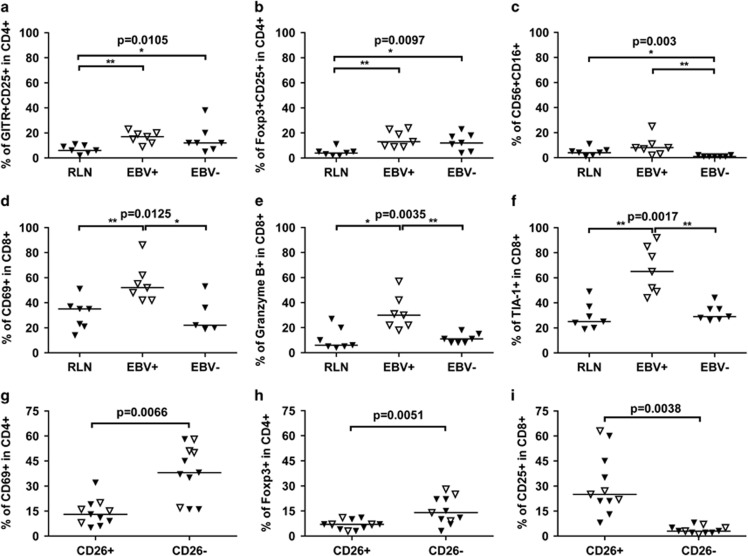
Significantly different immune cell populations in cHL. Optimized concentrations of fluorochrome-antibody conjugates were applied on cell suspensions of cHL tissue and analyzed by flow cytometry. (**a–f**) Significant differences in immune cell populations between RLN and EBV+ and EBV− cHL. Percentages of (**a**) GITR+CD25+ in CD4+ cells; (**b**) FoxP3+CD25+ in CD4+ cells; (**c**) CD16+ in CD56+ cells; (**d**) CD69+ in CD8+ cells; (**e**) Granzyme B+ in CD8+ cells; (**f**) TIA-1+ in CD8+ cells. An overview of all subsets tested and of all results has been given in Supplementary Tables S1–3. Lines indicate the median percentage of each group. Kruskal–Wallis test was used to test for differences in all three groups and a *P*-value of 0.0125 (to correct for multiple testing) was considered to be significant. Mann–Whitney *U* test was used as a *post hoc* test to identify significant differences in groups. (*P<0.05; **P<0.01). (**g**–**i**) Cell populations with significant differences between CD26− and CD26+ T cells in cHL overall and EBV-stratified subgroups. (**g**) Percentages of CD69+ in CD4+ cells in cHL; (**h**) FoxP3+ in CD4+ cells in cHL; (**i**) CD25+ in CD8+ cells in cHL. Lines indicate the median percentage of each group. Wilcoxon-matched-pairs signed-rank test was used to compare the two groups.

**Table 1:** Characteristics of the study cohorts

*Clinical characteristics*	*RLN* N=*7*	*All cHL* N=*14*	*EBV+ cHL* N=*7*	*EBV*− *cHL* N=*7*	P*-value*
*Gender*					
Male (%)	4 (57)	7 (50)	5 (71)	2 (29)	ns
*Age*					
Mean (range)	43 (17–72)	36 (12–76)	42 (12–76)	29 (13–54)	ns
*Histology subtype*					
NS (%)	—	8 (57)	3 (43)	5 (72)	
MC (%)	—	3 (21)	3 (43)	0	ns
LR (%)	—	2 (14)	1 (14)	1 (14)	
NOS (%)	—	1 (7)	0	1 (14)	

Abbreviations: cHL, classical Hodgkin lymphoma; EBV, Epstein–Barr virus; LR, lymphocyte-rich; MC, mixed cellularity; N, number; NOS, not otherwise specified; ns, not significant; NS, nodular sclerosis; RLN, reactive lymph node. Mann–Whitney *U* test was used to compare the differences between EBV+ cHL with EBV− cHL.
