# Supplementary material for: The microenvironment of classical Hodgkin lymphoma: heterogeneity by Epstein–Barr virus presence and location within the tumor
Source: Blood Cancer J. 2016 May 6;6(5):e417–. doi: 10.1038/bcj.2016.26 (PMC4916295; doi:10.1038/bcj.2016.26)
Supplement: Supplementary Information [file bcj201626x1.docx]

**Supplementary table S1. Overview of 46 cell subsets studied**

BD: BD Biosciences; IQ: IQ Products, Groningen, Netherlands; R&D: R&D systems, Minneapolis, MN USA; Dako: Dako Products, Glostrup, Denmark; BC: Beckman Coulter, Woerden, Netherlands.

**Supplementary Table S2. Statistical analysis of 46 cell populations**

K-W: Kruskal-Wallis Test; P values shown in bold indicate significant differences.

**Supplementary Table S3. Statistical analysis of 10 cell populations using CD26 to discriminate between cells outside and within tumor cell areas**

W-T: Wilcoxon matched-pairs signed rank test. P value shown in bold indicate significant differences. CD26+: outside tumor area; CD26-: surrounding the tumor.
